# Supplementary material for: Donor-strand exchange drives assembly of the TasA scaffold in Bacillus subtilis biofilms
Source: Nat Commun. 2022 Nov 18;13:7082. doi: 10.1038/s41467-022-34700-z (PMC9674648; doi:10.1038/s41467-022-34700-z)
Supplement: Supplementary file 1 — Supplementary_Information [file 41467_2022_34700_MOESM1_ESM.pdf]

Supplementary Information

Containing Figures S1-S9 and Tables S1-S3

"Donor-strand exchange drives assembly of the TasA scaffold in *Bacillus subtilis* biofilms"

Jan Böhning[1], Mnar Ghrayeb[2,3], Conrado Pedebos[4], Daniel K. Abbas[1], Syma Khalid[4], Liraz Chai[2,3,*], Tanmay A. M. Bharat[1,5,*]

[1] Sir William Dunn School of Pathology, University of Oxford, Oxford OX1 3RE, United Kingdom
[2] Institute of Chemistry, The Hebrew University of Jerusalem, Edmond J. Safra Campus, Jerusalem 91904, Israel
[3] The Center for Nanoscience and Nanotechnology, The Hebrew University of Jerusalem, Edmond J. Safra Campus, Jerusalem 91904, Israel
[4] Department of Biochemistry, University of Oxford, Oxford OX1 3QU, UK
[5] Structural Studies Division, MRC Laboratory of Molecular Biology, Francis Crick Avenue, Cambridge CB2 0QH, United Kingdom

* Correspondence: liraz.chai@mail.huji.ac.il and tbharat@mrc-lmb.cam.ac.uk

Supplementary Figures S1-S9

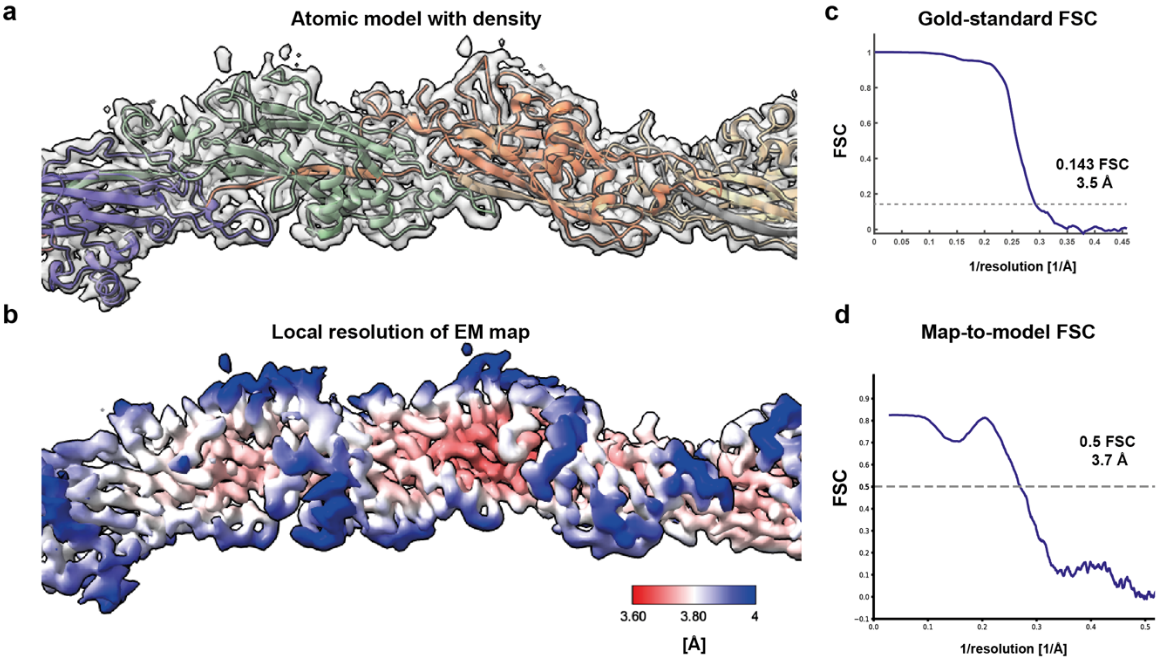

Figure S1: Cryo-EM structure of TasA fibres. a) TasA fibre atomic model, shown as differently coloured ribbon diagrams, within the cryo-EM density shown at 12 σ isosurface contour level. **b)** Local resolution estimation of the cryo-EM reconstruction. **c)** Masked gold-standard Fourier Shell Correlation (FSC) plot. **d)** Map-to-model FSC curve.

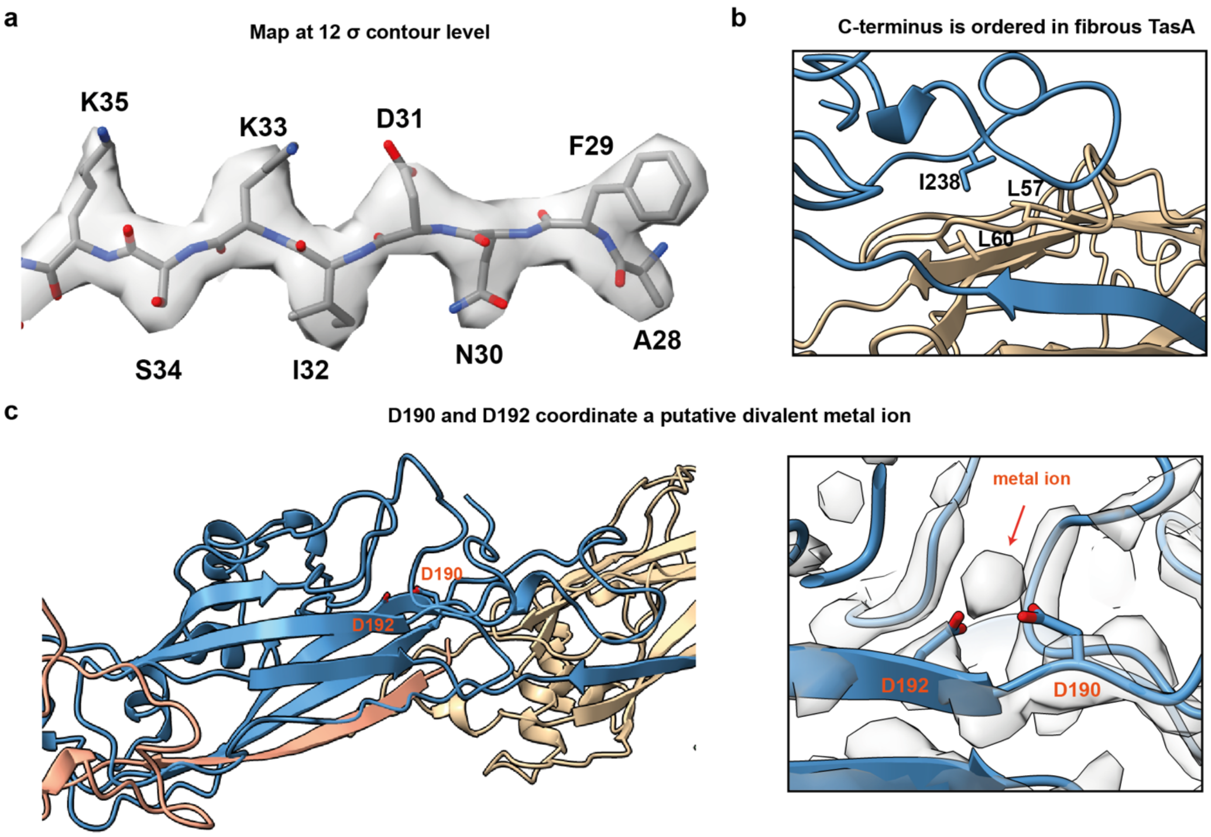

Figure S2: Structural features of TasA. a) Map at a 12 σ contour level with atomic model of TasA N-terminal residues shown. **b)** Interaction of hydrophobic residue I238 of the n_{th} subunit (blue ribbon) within the C-terminus, with the $(n+1)_{th}$ subunit (beige ribbon). **c)** A putative cation co-ordination site is resolved in the cryo-EM map. While cryo-EM does not allow determining the chemical identity of the ion, TasA shows homology to the camelysin CalY in *B. cereus*, which was found to contain a zinc ion[1].

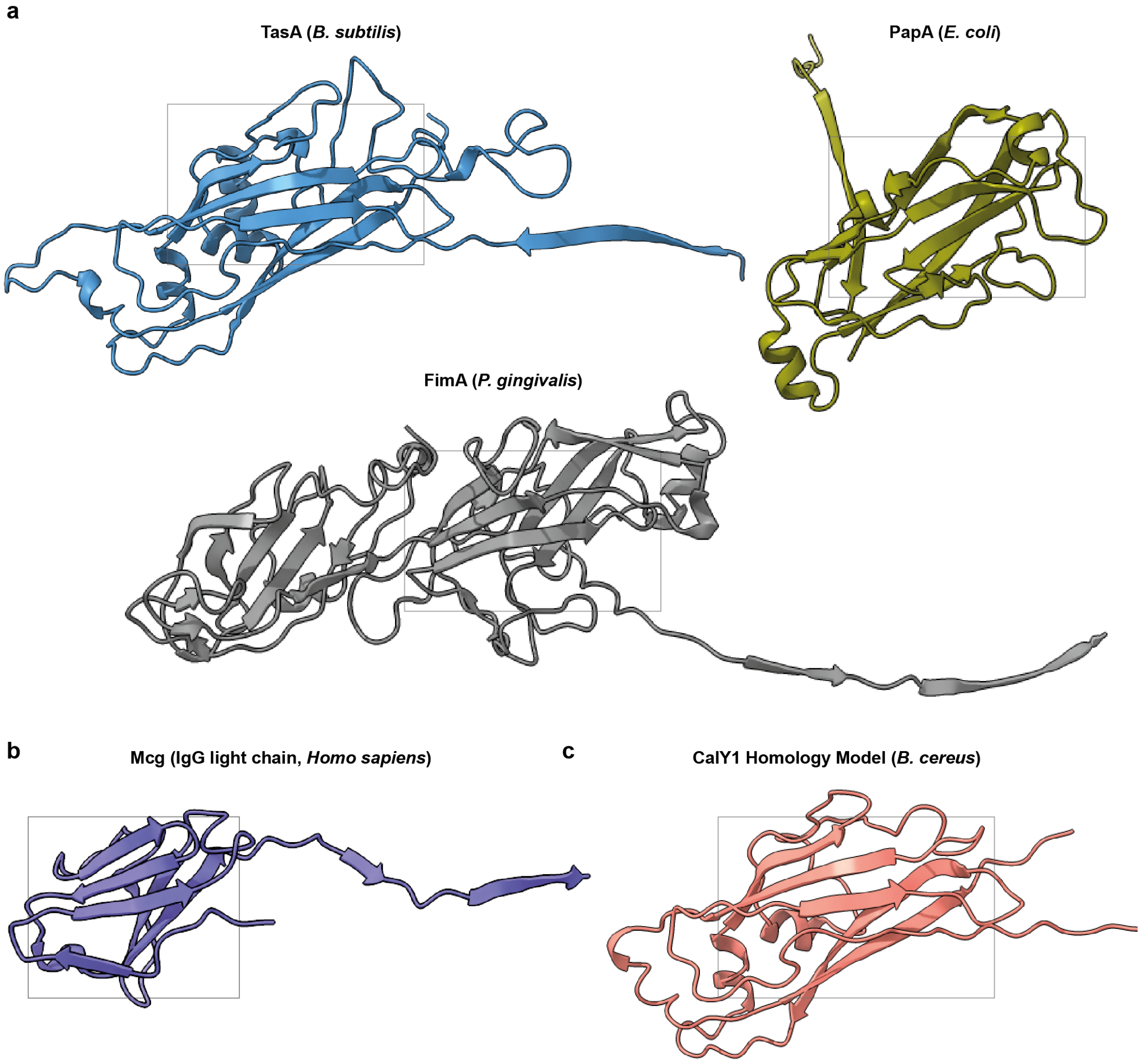

Figure S3: Comparison of the TasA structure (fibre form) with proteins from other systems undergoing donor-strand complementation. a) Comparison of TasA (blue ribbon) with the PapA Chaperone-Usher Pathway pilin of a type I pilus from *Escherichia coli*[2] (PDB 5FLU, military green ribbon) and the FimA pilin of a type V pilus from *Porphyromonas gingivalis*[3] (PDB 6KMF, grey ribbon). **b)** Donor-strand-complemented structure of the Mcg IgG light chain protein involved in amyloidosis[4]. **c)** Based on the atomic structure of TasA, we also created a homology model of an accessory protein called CalY1 which is encoded in the same operon as TasA in *B. cereus*[1,5]. Given significant sequence homology to TasA and conservation of hydrophobic residues involved in donor-strand complementation, CalY1 might also polymerise using a donor-strand mechanism. These comparisons suggest that a β-sandwich (grey boxes) fold is a common structural feature of proteins undergoing donor-strand complementation.

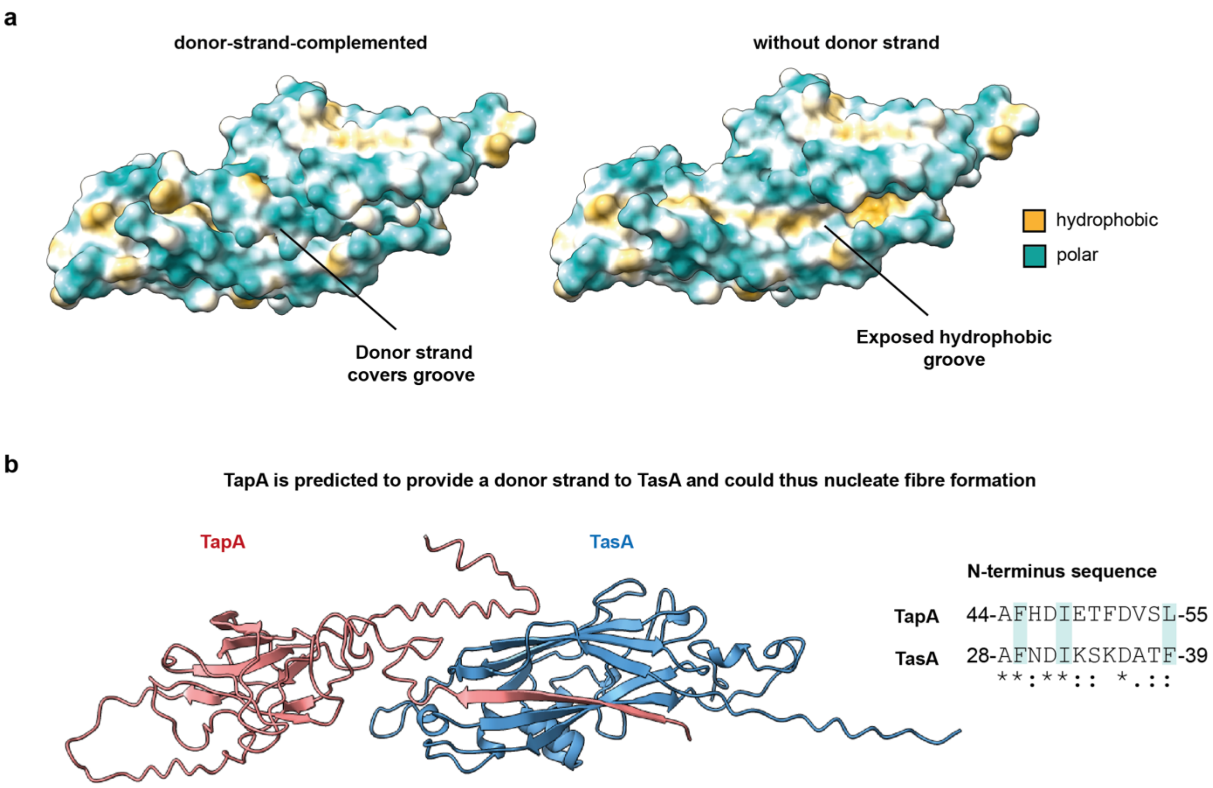

Figure S4: TapA is predicted to extend a donor strand that complements TasA. a) The donor strand shields a hydrophobic groove in TasA (left), which is exposed in the absence of the donor strand (right). **b)** AlphaFold 2 multimer prediction of a TapA/TasA complex. A Clustal Omega alignment of N-terminal residues of TapA and TasA is shown on the upper right; the hydrophobic residues mediating major donor strand interactions with the complemented subunit (see Figure 1) are marked in cyan. Asterisks indicate full conservation, colons indicate conservation between groups of strongly similar properties, and periods indicate conservation between groups of weakly similar properties as per Clustal Omega output.

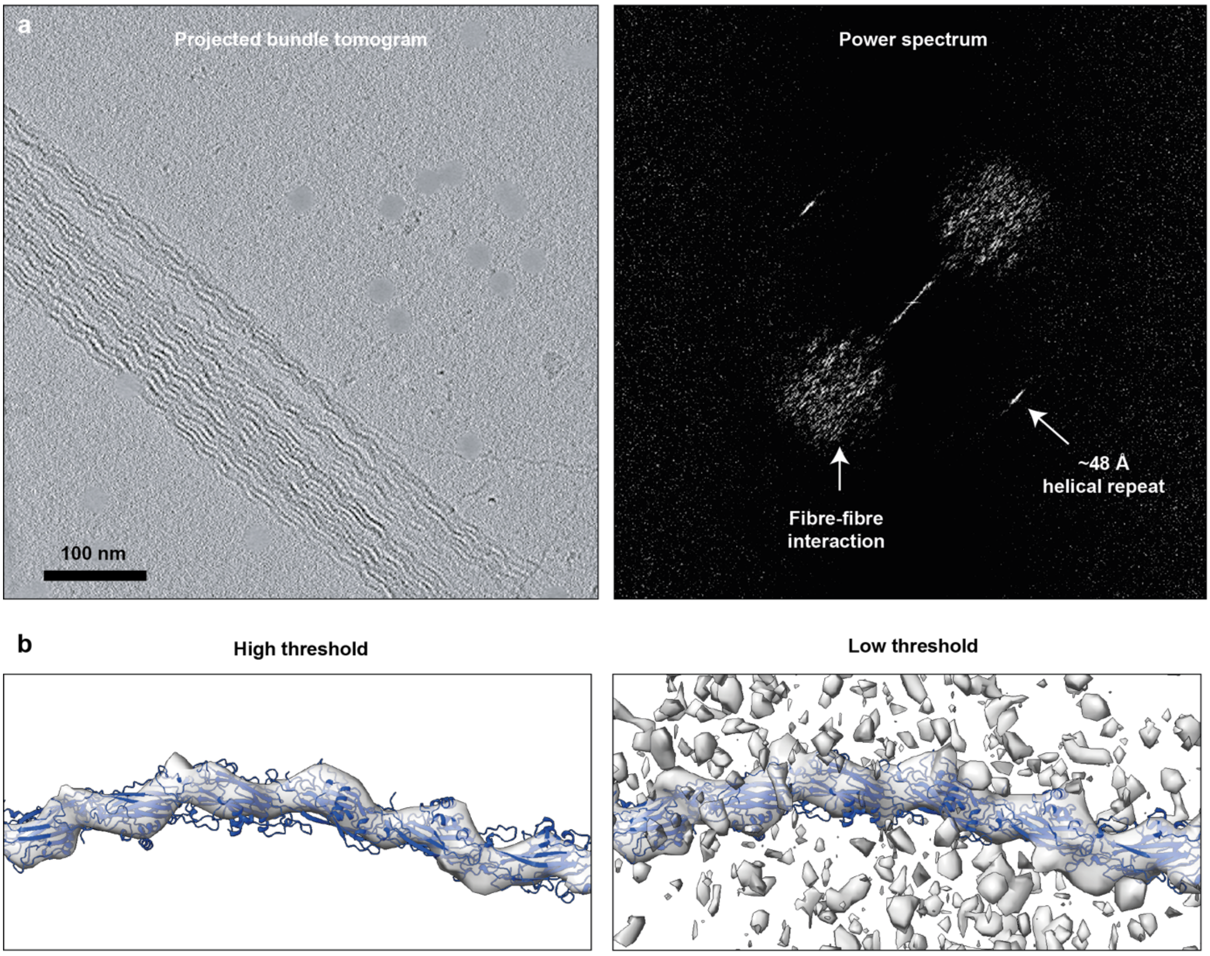

Figure S5: Interactions between TasA fibres are non-rigid. a) Left: Projected tomogram of a purified TasA bundle. Right: Power spectrum of the Fourier transform showing a distinct layer line for 48 Å (single TasA subunit repeat) but no distinct spots for interactions between fibres. **b)** Unmasked subtomogram averages of TasA fibres picked in bundles. While the architecture of individual TasA fibres can be resolved, no interacting fibres could be detected.

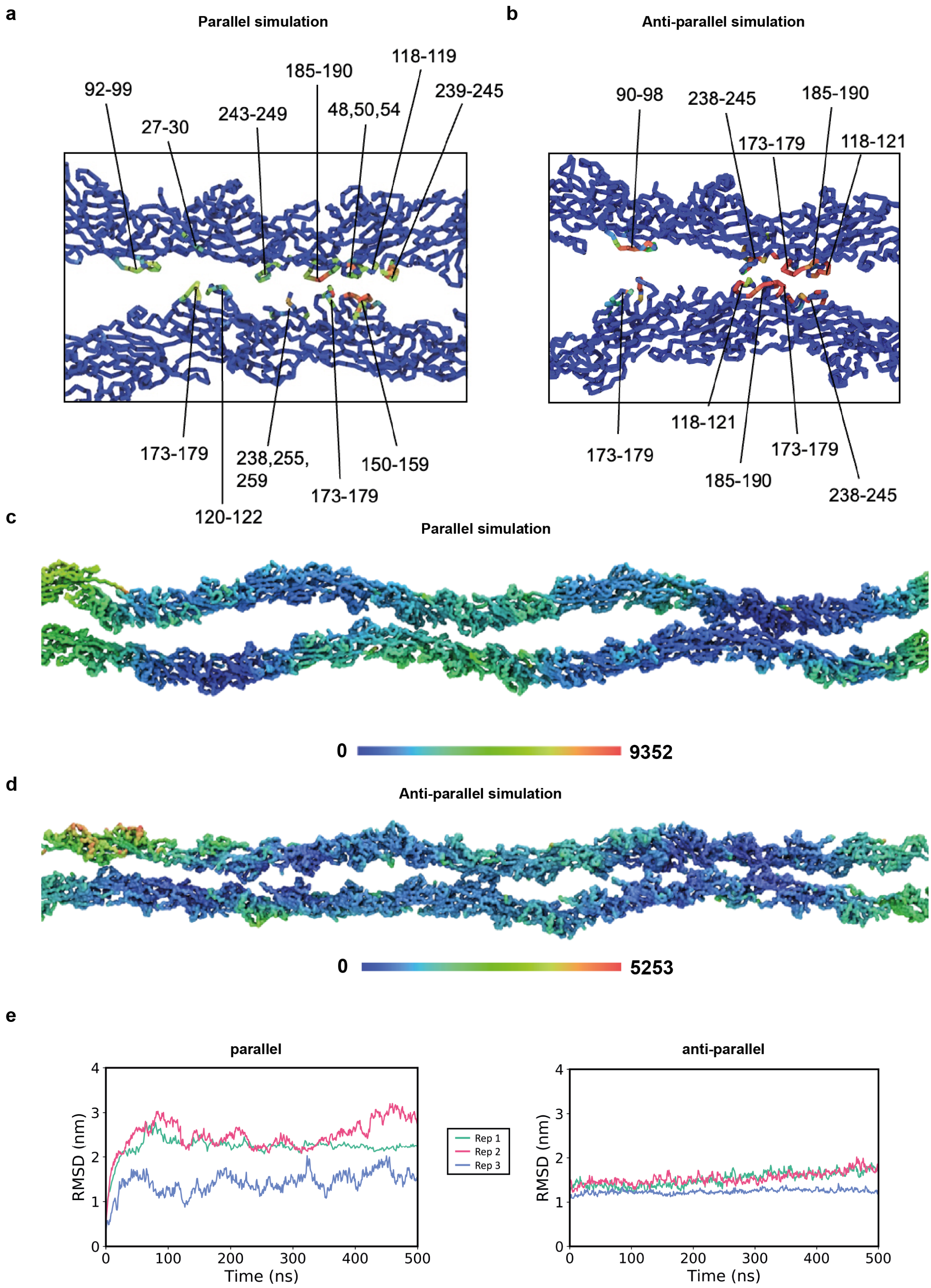

Figure S6: MD simulations of TasA doublets. a) Stable solution of coarse-grained MD (500 ns) of two TasA fibres interacting in parallel. Residues found interacting during the simulation at the inter-fibre interface are indicated; red: strong interactions, green, weak interactions. **b)** Stable solution as in a) but for an antiparallel interaction.

c) Parallel two-fibre solution zoomed out with B-factor colour-plotted (in $Å^2$), obtained by converting the RMSF data from the simulations. **d)** Anti-parallel two-fibre model zoomed out with with B-factor colour-plotted (in $Å^2$), obtained by converting the RMSF data from the simulations. **e)** Root mean square deviation (RMSD) of parallel and antiparallel simulations.

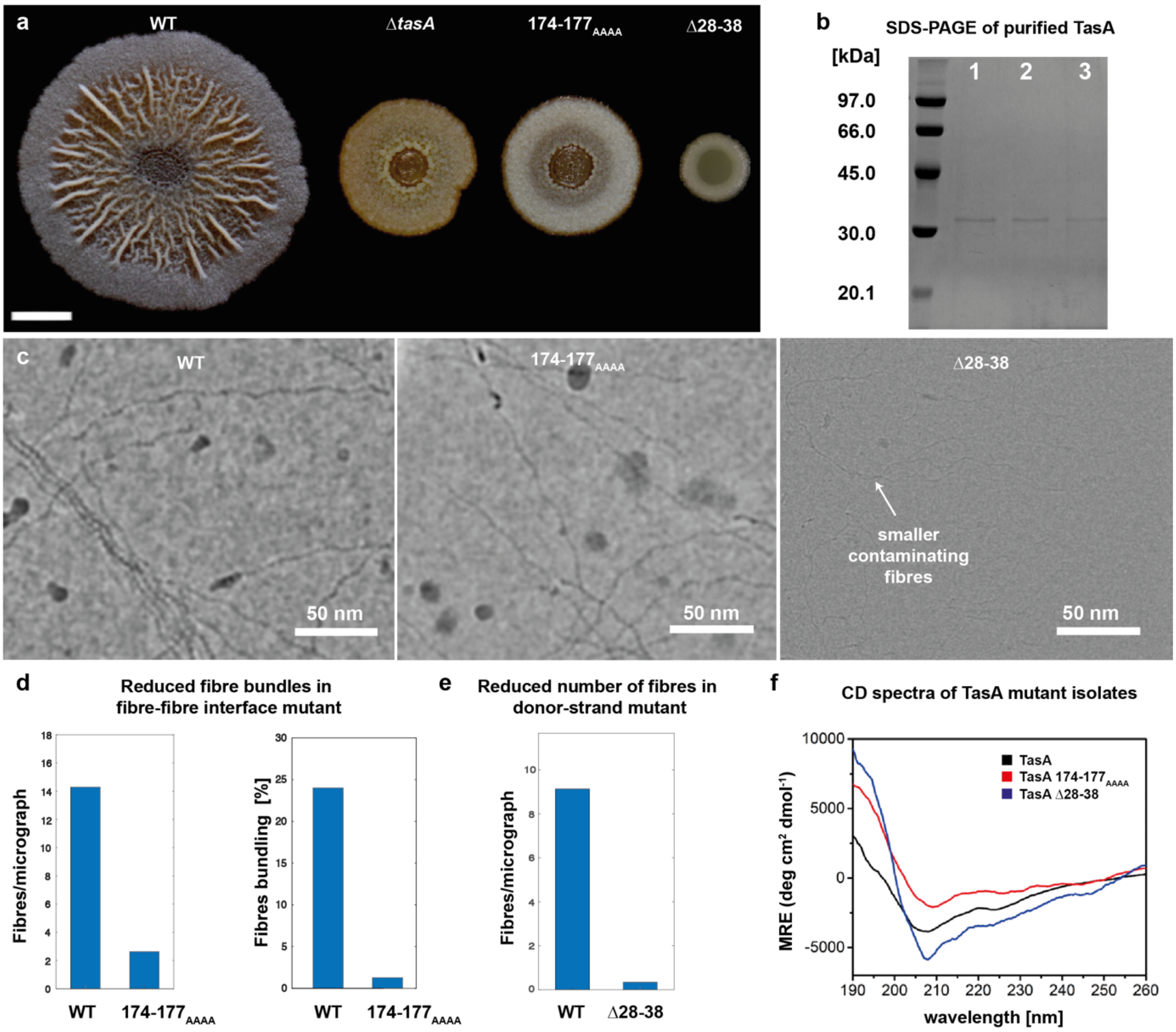

Figure S7: Mutational studies of TasA. a) *B. subtilis* biofilms grown on agar plates, imaged after 72 hours post inoculation. From left to right: wild-type (WT, NCIB 3610), Δ*tasA* (ZK3657), *tasA* 174-177$_{AAAA}$ (MG4, fibre-fibre interface mutant) and *tasA* Δ28-38 (MG3, donor-strand mutant). Scale bar corresponds to 0.5 cm. **b)** SDS-PAGE of TasA protein isolates. Lane 1: TasA (unmutated), lane 2: TasA Δ28-38, lane 3: TasA 174-177$_{AAAA}$, **c)** cryo-EM images comparing fibres of TasA purified from the Δ*sinR* Δ*eps* strain (ZK4363, see Methods, left). In a fibre-fibre interface mutant TasA (174-177$_{AAAA}$ TasA, purified from MG2, middle), bundling is significantly reduced. A donor-strand mutant TasA (Δ28-38 TasA, purified from MG1, right) shows a markedly reduced number of TasA fibres, while smaller aberrant, contaminating fibres can be faintly seen instead. All specimens are at the same measured protein concentrations. **d)** Quantification of bundling behaviour and fibre number per micrograph in the fibre-fibre interface mutant TasA (174-177$_{AAAA}$). **e)** Quantification of TasA fibres in cryo-EM images of donor-strand mutant TasA (Δ28-38). **f)** Circular Dichroism spectra of unmutated TasA, 174-177$_{AAAA}$ TasA and Δ28-38 TasA purified from a Δ*sinR* Δ*eps* strain).

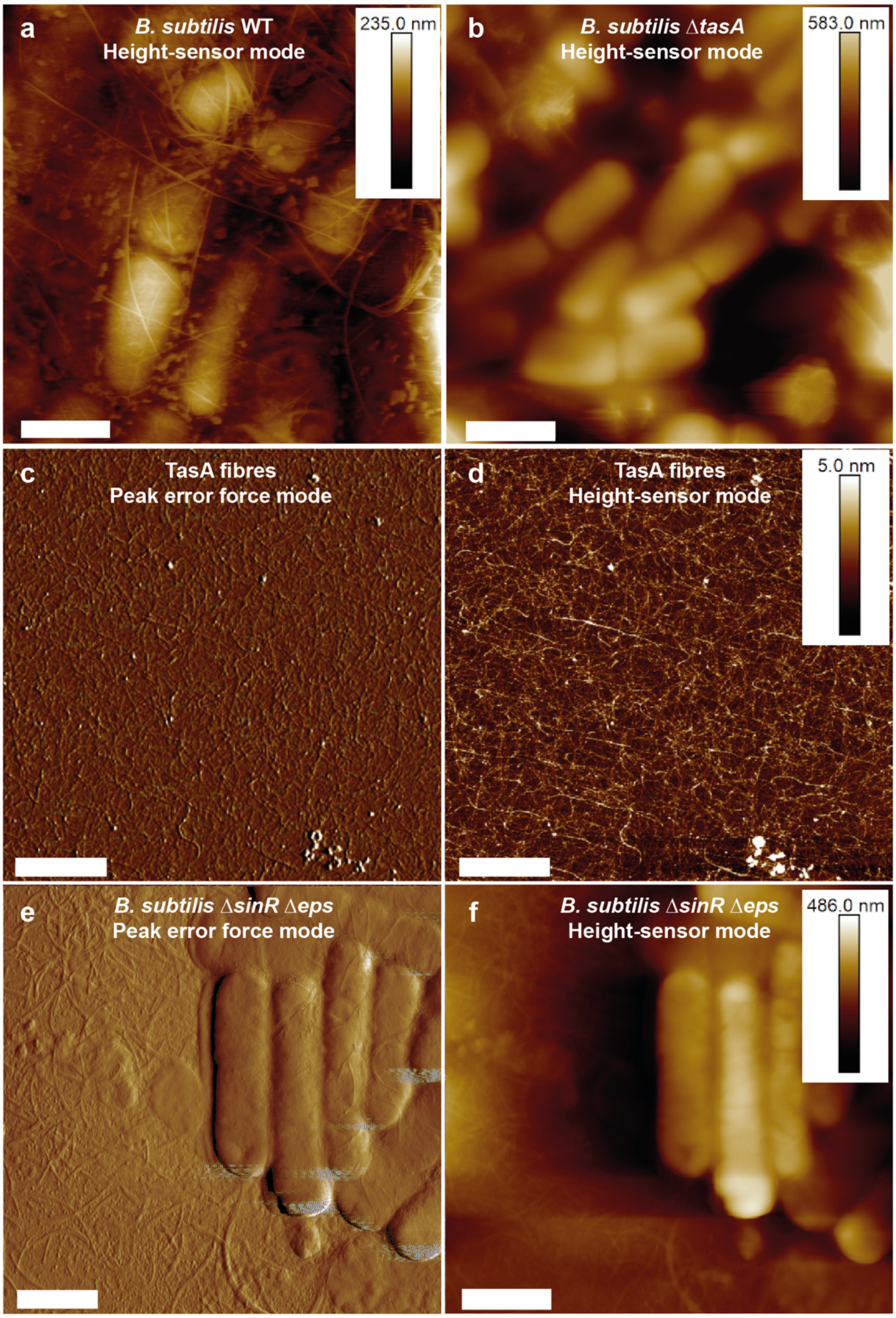

Figure S8: AFM imaging of *B. subtilis* biofilms and purified TasA fibres. a-b) Height sensor mode AFM images of a) WT (ZK5041) and b) Δ*tasA* (ZK3657) pellicles. **c)** AFM peak force error and **d)** height sensor mode AFM images of TasA fibres formed *in vitro* from purified protein. Height scale is shown to the top-right of each relevant image. **e)** Peak Force error and **f)** Height-Sensor mode AFM images of Δ*sinR* Δ*eps* biofilms (ZK4363). Scale bars are 1 µm. We had furthermore performed fluorescence microscopy assays using pellicles from a previously generated strain that expresses

a TasA-mCherry fusion[6] (data not shown). However, following the reviewers' suggestion, we removed those assays from the final version, as it was brought to our attention that the TasA-mCherry strain appears to produce free TasA in addition to full-length TasA-mCherry, which suggests that the strain's phenotype cannot be conclusively linked with the TasA-mCherry form of the protein (see pages 45-47 of the peer review file published with ref[7] at https://www.nature.com/articles/s41467-020-15758-z#Sec32).

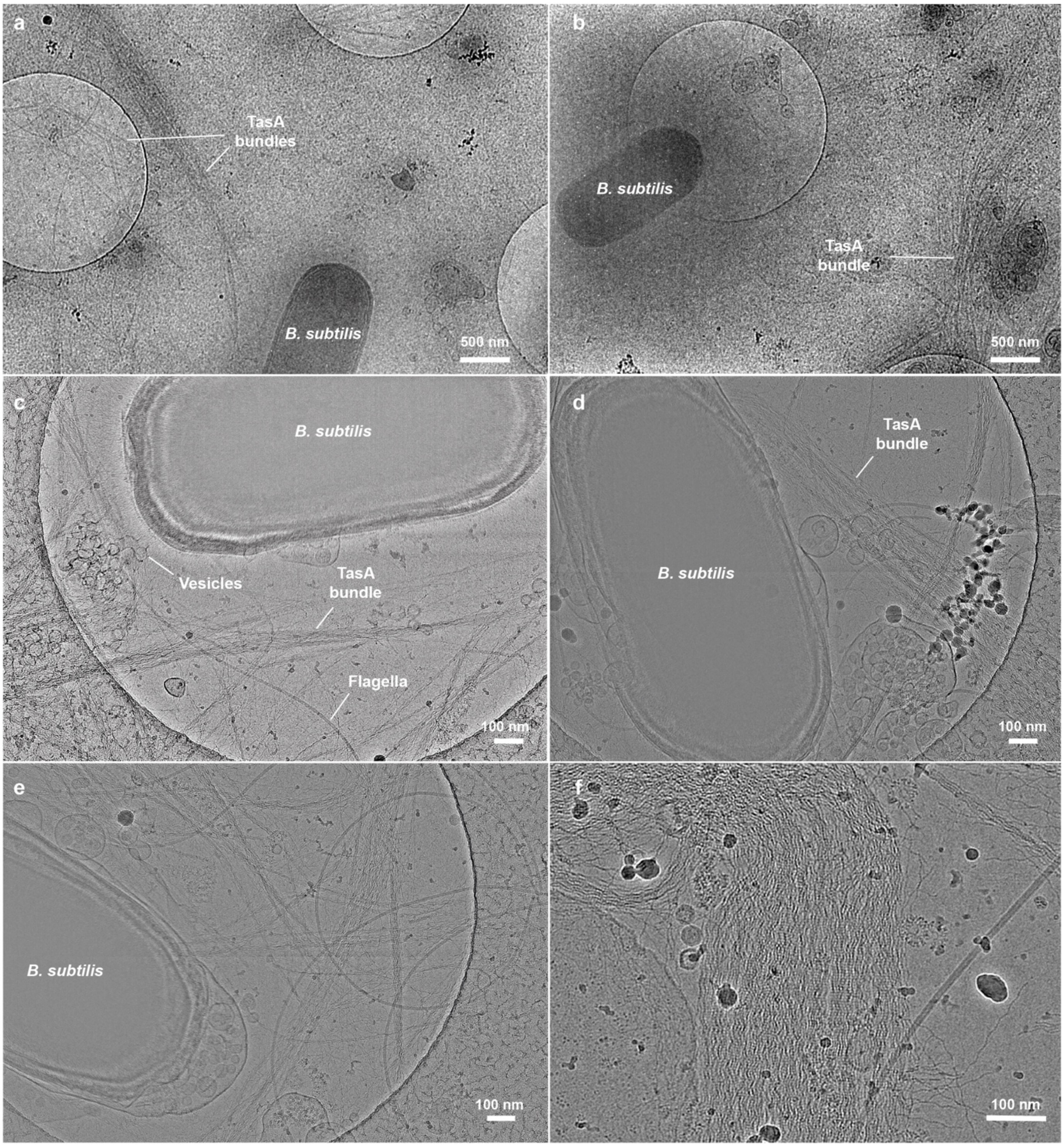

Figure S9: Cryo-EM of the ECM components of *B. subtilis* ∆*sinR* ∆*eps* biofilms. a-f) Gallery of medium- and high magnification images of resuspended *B. subtilis* ∆*sinR* ∆*eps* biofilms showing TasA bundles surrounding *B. subtilis* cells.

Supplementary Tables S1-S3

Table S1. TasA cryo-EM data acquisition and processing statistics.

**Data collection and processing**	EMDB-15673
Magnification	81,000
Voltage (kV)	300
Electron exposure (e–/Å^2)	48.5
Defocus range (μm)	-1 to -2
Pixel size (Å)	0.546 (super-resolution)
Symmetry imposed	Helical, final: -55.18°, 48.36 Å
Initial particle images (no.)	1,478,370
Final particle images (no.)	103,009
Map resolution (Å)	3.5 (0.143)
FSC threshold	
Map resolution range (Å)	3.6 – 4.1
**Refinement**	PDB-8AUR
Initial model used (PDB code)	5OF1
Model resolution (Å)	3.7 (0.5)
FSC threshold	
Model resolution range (Å)	n/a
Map sharpening *B* factor (Å^2)	-67.9
Model composition	
Non-hydrogen atoms	5,409
Protein residues	699
Ligands	0
B factors (Å^2)	
Protein	
Ligand	
R.m.s. deviations	
Bond lengths (Å)	0.002
Bond angles (°)	0.544
Validation	
MolProbity score	1.73
Clashscore	6.34
Poor rotamers (%)	0
Ramachandran plot	
Favored (%)	94.37
Allowed (%)	5.63
Disallowed (%)	0

Table S2. List of primers used in this study.

**Δ28-38 TasA**	Primers sequences
T2838P1 forward	5’ GTATATAAACCGGCAGGCTACCCG 3’
T2838P2 reverse	5’ CGTACCTGATGCAAAAGTTGCCCATGTTCCTCCTCCAAC 3’
T2838P3 forward	5’ ACTTTTGCATCAGGTACGCTTG 3’
T2838P4 reverse	5’ CTGAGCGAGGGAGCAGAATTAATTTTTATCCTCGCTATGCGCTTTTTC 3’
T2838P5 forward	5’ GTTGACCAGTGCTCCCTGTAACAGCAAAAAAAAGAGACGGCC 3’
T2838P6 reverse	5’ AGTTGAAATTGAAAATGGCGGATTGTTT 3’
**Δ174-177$_{AAAA}$**	
T174AP1forward	5’ GACGTTATTACATTTATGCAGGATGCAAAT 3’
T174AP2 reverse	5’ AGGAGCTGCAGCTGCAGCAATTGTTGCTACATTGACTTTACCGCTTG 3’
T174AP3 forward	5’ GCTGCAGCTGCAGCTCCTGAATATGATGGTGTTCCA 3’
**Sequencing**	
PseqF Forward	5’ ACAAAGGACAGCACCATGTC TA 3’
PseqR Reverse	5’ CTGAGCGAGGGAGCAGAATTAATTTTTATCCTCGCTATGCGCTTTTTC 3’

Table S3. List of *B. subtilis* strains used in this study.

**Strain**	**Genotype**	**Reference**
NCIB 3610	Wild-type, undomesticated strain	Branda et al., (2001)[8]
ZK4363	NCIB 3610 yve-yvf::tet (epsA-O::tet) sinR::spc	Romero et al., (2010)[9]
ZK3657	NCIB N3610 tasA::kan	Vlamakis et., (2008)[10]
ZK5041	NCIB 3610 tasAop-mCherry (from a previous study[6])	Kolodkin-Gal et al., (2010)[6]
MG1	NCIB 3610 yve-yvf::tet (epsA-O::tet) sinR::spc tasA$^{\Delta 28\text{-}38}$-kan	This study
MG2	NCIB 3610 yve-yvf::tet (epsA-O::tet) sinR::spc<br>tasA-D174A, G175A, K176A, T177A-kan	This study
MG3	NCIB 3610 tasA$^{\Delta 28\text{-}38}$-kan	This study
MG4	NCIB 3610 tasA-D174A, G175A, K176A, T177A-kan	This study

Supplementary References

1 Grass, G. *et al.* Camelysin is a novel surface metalloproteinase from *Bacillus cereus*. *Infection and Immunity* **72**, 219-228 (2004).

2 Hospenthal, M. K. *et al.* Structure of a chaperone-usher pilus reveals the molecular basis of rod uncoiling. *Cell* **164**, 269-278 (2016).

3 Shibata, S. *et al.* Structure of polymerized type V pilin reveals assembly mechanism involving protease-mediated strand exchange. *Nature Microbiology* **5**, 830-837 (2020).

4 Brumshtein, B. *et al.* Formation of amyloid fibers by monomeric light chain variable domains. *Journal of Biological Chemistry* **289**, 27513-27525 (2014).

5 Caro-Astorga, J., Pérez-García, A., de Vicente, A. & Romero, D. A genomic region involved in the formation of adhesin fibers in *Bacillus cereus* biofilms. *Frontiers in Microbiology* **5**, 745 (2015).

6 Kolodkin-Gal, I. *et al.* D-amino acids trigger biofilm disassembly. *Science* **328**, 627-629 (2010).

7 Cámara-Almirón, J. *et al.* Dual functionality of the amyloid protein TasA in *Bacillus* physiology and fitness on the phylloplane. *Nature Communications* **11**, 1-21 (2020).

8 Branda, S. S., González-Pastor, J. E., Ben-Yehuda, S., Losick, R. & Kolter, R. Fruiting body formation by *Bacillus subtilis*. *Proceedings of the National Academy of Sciences* **98**, 11621-11626 (2001).

9 Romero, D., Aguilar, C., Losick, R. & Kolter, R. Amyloid fibers provide structural integrity to Bacillus subtilis biofilms. *Proceedings of the National Academy of Sciences* **107**, 2230-2234 (2010).

10 Vlamakis, H., Aguilar, C., Losick, R. & Kolter, R. Control of cell fate by the formation of an architecturally complex bacterial community. *Genes Dev* **22**, 945-953, doi:10.1101/gad.1645008 (2008).
