# Supplementary material for: Donor-strand exchange drives assembly of the TasA scaffold in Bacillus subtilis biofilms
Source: Nat Commun. 2022 Nov 18;13:7082. doi: 10.1038/s41467-022-34700-z (PMC9674648; doi:10.1038/s41467-022-34700-z)
Supplement: Supplementary file 3 — Description of Additional Supplementary Files [file 41467_2022_34700_MOESM3_ESM.pdf]

Description of Additional Supplementary Files:

Movie S1: Atomic structure of TasA. Density and ribbon depiction of the atomic model of TasA fibres demonstrating extensive subunit interactions provided by an N-terminal donor strand.

Movie S2: Comparison of monomeric with fibrous TasA. Monomeric TasA (PDB 5OF2) is compared to the TasA fibre state (this study), employing a structural morph as calculated in ChimeraX to demonstrate structural differences between the states.

Movie S3: Electron cryotomography of TasA bundles. Shown are sequential slices of a tomogram of TasA bundles.

Movie S4: Molecular dynamics simulation of a TasA fibre doublet. Length of the simulation is 200 ns.
