# Supplementary material for: Donor-strand exchange drives assembly of the TasA scaffold in Bacillus subtilis biofilms
Source: Nat Commun. 2022 Nov 18;13:7082. doi: 10.1038/s41467-022-34700-z (PMC9674648; doi:10.1038/s41467-022-34700-z)
Supplement: Supplementary file 8 — Reporting Summary [file 41467_2022_34700_MOESM8_ESM.pdf]

nature portfolio

Corresponding author(s): Tanmay Bharat, Liraz Chai

Last updated by author(s): 26/10/22

Reporting Summary

Nature Portfolio wishes to improve the reproducibility of the work that we publish. This form provides structure for consistency and transparency in reporting. For further information on Nature Portfolio policies, see our Editorial Policies and the Editorial Policy Checklist.

Statistics

For all statistical analyses, confirm that the following items are present in the figure legend, table legend, main text, or Methods section.

n/a	Confirmed	
☐	☒	The exact sample size (*n*) for each experimental group/condition, given as a discrete number and unit of measurement
☐	☒	A statement on whether measurements were taken from distinct samples or whether the same sample was measured repeatedly
☒	☐	The statistical test(s) used AND whether they are one- or two-sided<br>*Only common tests should be described solely by name; describe more complex techniques in the Methods section.*
☒	☐	A description of all covariates tested
☒	☐	A description of any assumptions or corrections, such as tests of normality and adjustment for multiple comparisons
☐	☒	A full description of the statistical parameters including central tendency (e.g. means) or other basic estimates (e.g. regression coefficient) AND variation (e.g. standard deviation) or associated estimates of uncertainty (e.g. confidence intervals)
☒	☐	For null hypothesis testing, the test statistic (e.g. *F*, *t*, *r*) with confidence intervals, effect sizes, degrees of freedom and *P* value noted<br>*Give P values as exact values whenever suitable.*
☒	☐	For Bayesian analysis, information on the choice of priors and Markov chain Monte Carlo settings
☒	☐	For hierarchical and complex designs, identification of the appropriate level for tests and full reporting of outcomes
☒	☐	Estimates of effect sizes (e.g. Cohen's *d*, Pearson's *r*), indicating how they were calculated

Our web collection on statistics for biologists contains articles on many of the points above.

Software and code

Policy information about availability of computer code

Data collection	EPU 2.9, SerialEM 3.8, Nanoscope Analysis 2.0, NIS-Elements AR 4.20
Data analysis	RELION 3.1, RELION 4.0, COOT 0.9, PHENIX 1.20, Nanoscope Analysis 2.0, OriginPro 2022 SR1, NIS-Elements AR 4.20, MATLAB R2021b, MotionCor2, CtfFind4, UCSF ChimeraX 1.3, COOT 0.9

For manuscripts utilizing custom algorithms or software that are central to the research but not yet described in published literature, software must be made available to editors and reviewers. We strongly encourage code deposition in a community repository (e.g. GitHub). See the Nature Portfolio guidelines for submitting code & software for further information.

Data

Policy information about availability of data

All manuscripts must include a data availability statement. This statement should provide the following information, where applicable:

- Accession codes, unique identifiers, or web links for publicly available datasets
- A description of any restrictions on data availability
- For clinical datasets or third party data, please ensure that the statement adheres to our policy

The cryo-EM density map of a TasA fibre generated in this study has been deposited in the Electron Microscopy Databank (EMDB) under the accession number EMD-15673. The corresponding atomic coordinates are deposited in the Protein Data Bank (PDB) under accession code 8AUR. The atomic coordinates of monomeric TasA used for model building and structural comparison are available in the PDB under accession code 5OF1 and 5OF2. The atomic coordinates of proteins used for structural comparison with TasA in Figure S3 are available in the PDB under accession codes 5FLU (PapA), 6KMF (FimA) and 4UNT (Mcg). All other

data are available from the corresponding authors upon request.

Human research participants

Policy information about studies involving human research participants and Sex and Gender in Research.

Reporting on sex and gender	There were no human research participants in this study.
Population characteristics	There were no human research participants in this study.
Recruitment	There were no human research participants in this study.
Ethics oversight	There were no human research participants in this study.

Note that full information on the approval of the study protocol must also be provided in the manuscript.

Field-specific reporting

Please select the one below that is the best fit for your research. If you are not sure, read the appropriate sections before making your selection.

[x] Life sciences [ ] Behavioural & social sciences [ ] Ecological, evolutionary & environmental sciences

For a reference copy of the document with all sections, see nature.com/documents/nr-reporting-summary-flat.pdf

Life sciences study design

All studies must disclose on these points even when the disclosure is negative.

Sample size	Sample sizes were not calculated; there are no statistical determinations or tests performed within this study.
Data exclusions	Data was excluded as part of cryo-EM workflows where low-quality particles that did not support the solution of a high-resolution structure are discarded, in agreement with typical cryo-EM classification schemes.
Replication	Number of replicates are listed within the Statistics and Reproducibility section, tables or Methods where appropriate.
Randomization	Randomisation of datasets was not employed as randomisation is not typically applied for cryo-EM and AFM workflows, with the exception for random assignments of particles into half-sets during refinement as part of typical cryo-EM workflows to allow gold-standard resolution estimation.
Blinding	Blinding was not applied to this study as it is not typically applied in cryo-EM or AFM techniques.

Reporting for specific materials, systems and methods

We require information from authors about some types of materials, experimental systems and methods used in many studies. Here, indicate whether each material, system or method listed is relevant to your study. If you are not sure if a list item applies to your research, read the appropriate section before selecting a response.

Materials & experimental systems

n/a	Involved in the study	
[x]	[ ]	Antibodies
[x]	[ ]	Eukaryotic cell lines
[x]	[ ]	Palaeontology and archaeology
[x]	[ ]	Animals and other organisms
[x]	[ ]	Clinical data
[x]	[ ]	Dual use research of concern

Methods

n/a	Involved in the study	
[x]	[ ]	ChIP-seq
[x]	[ ]	Flow cytometry
[x]	[ ]	MRI-based neuroimaging
